# Analysis of curative effect of percutaneous coaxial large channel endoscopic lumbar interbody fusion in the treatment of degenerative lumbar spinal stenosis

**DOI:** 10.3389/fsurg.2022.1002734

**Published:** 2022-10-05

**Authors:** Pin Feng, Qingquan Kong, Bin Zhang, Junlin Liu, Junsong Ma, Yuan Hu

**Affiliations:** ^1^ Department of Orthopedics Surgery, West China Hospital, Sichuan University, Chengdu, China; ^2^ Department of Orthopedics Surgery, Hospital of Chengdu Office of People's Government of Tibetan Autonomous Region, Chengdu, China

**Keywords:** percutaneous endoscopic, lumbar interbody fusion, coaxial large channel, lumbar spinal stenosis, curative effect

## Abstract

**Objective:**

To investigate the clinical efficacy and technical points of Percutaneous Coaxial Large-channel Endoscopic Lumbar Interbody Fusion (PCLE-LIF) in the treatment of degenerative lumbar spinal stenosis.

**Methods:**

The clinical data of patients with single-segment degenerative lumbar spinal stenosis who underwent PCLE-LIF surgery from January 2019 to June 2021 were retrospectively analyzed. Surgery-related data included symptom duration, operation time, hospital stay, and complication rate. Functional score data included low back pain and lower extremity pain VAS score, ODI score, and MacNab criteria were used to evaluate clinical effects. The Brantigan criteria were used to evaluate the interbody fusion.

**Results:**

There were 62 patients in this group, including 35 males and 27 females. The surgical sites were all lower lumbar spine, including 35 cases of lumbar L4/5 and 27 cases of L5/S1. The length of hospital stay was 7.7 ± 1.4 days. All patients were followed up regularly for 1 year. The interbody fusion rate was 93.5% at 1 year after operation. There were 2 cases of numbness, 2 cases of nerve edema and pain, 1 case of cage displacement, and 1 case of pedicle screw loosening. The complication rate was 9.6%. The VAS scores of low back pain 1 day before surgery, 3 days, 3 months and 1 year after surgery were 4.48 ± 1.06, 0.84 ± 0.81, 0.40 ± 0.56, 0.39 ± 0.69, and the VAS of lower extremity pain at each time point of appeal were 5.58 ± 0.98, 0.91 ± 0.58, 0.31 ± 0.46, 0.19 ± 0.40. The ODI scores at 1 day before surgery, 3 months and 1 year after surgery were 60.01 ± 6.21, 15.58 ± 2.84, 8.82 ± 2.15. The ODI scores and VAS scores of low back pain and lower extremity pain at each follow-up time point after operation were significantly lower than those before operation (*p* < 0.05). The 1-year follow-up after operation was evaluated by the modified MacNab standard, and the results were excellent in 36 cases, good in 23 cases, fair in 3 cases, and poor in 0 cases, with an excellent and good rate of 95.2%.

**Conclusion:**

Percutaneous coaxial large-channel endoscopic lumbar interbody fusion in the treatment of degenerative lumbar spinal stenosis has good short-term efficacy and high safety, and is worthy of popularization.

## Background

Percutaneous endoscopic lumbar spine fusion technique was first reported by Leu (1) in 1996, but due to the high complication rate reported at that time and the backwardness of technology and surgical instrument, the technique has not been widely promoted and applied (2). In recent years, with the improvement of surgical instruments and the advancement of technology, the technology has regained the attention of minimally invasive spine surgeons. Most of the approaches used in the early literature reports were transforaminal approaches. Although the clinical efficacy was satisfactory, both the large-channel endoscopy system and the small-channel endoscopy system have shortcomings such as limited decompression range and outlet root injury (3, 4). The translaminar approach can achieve more adequate dorsal decompression, have a wider range of indications, and can effectively avoid damage to the outlet root. In recent literature reports, the translaminar space approach mainly adopts the dual-channel endoscopic system, which can achieve the same surgical effect and operation time as Mis-TLIF, but compared with the single-channel endoscopic system, the technology has Greater soft tissue injury and postoperative epidural hematoma incidence (5, 6), while the coaxial small channel endoscopy system is less clinically used due to low surgical efficiency. With the improvement of the instruments, the percutaneous coaxial large-channel endoscopy system has also been applied to the translaminar space approach, which can avoid the insufficiency of the dual-channel endoscopy system while ensuring the efficiency of the operation, but there is no relevant literature at present. In January 2019, our team began to use Percutaneous Coaxial Large-channel Endoscopic Lumbar Interbody Fusion (PCLE-LIF) for the treatment of degenerative lumbar spinal stenosis. and accumulated some clinical experience. This is a retrospective analysis to explore the clinical effect of PCLE-LIF in the treatment of degenerative lumbar spinal stenosis. The report is as follows:

## Methods and materials

From January 2019 to June 2021, 62 patients with single segment lumbar spinal stenosis diagnosed in our hospital who underwent PCLE-LIF surgery were included in this trial. The duration of symptoms, operation time, hospital stay and other general information of the patients were recorded. VAS scores of low back pain and lower extremity pain were compared and analyzed on the day before operation and 3 days, 3 months and 1 year after operation, and ODI scores were compared on the day before operation and 3 months and 1 year after operation, so as to evaluate the improvement of symptoms, and the clinical effect was evaluated by macnab standard. Interbody fusion was evaluated by brantigan standard one year after operation. According to the degree of fusion, it was divided into 1–5 levels, of which 4 and 5 were successful fusion, and 1, 2 and 3 were non fusion (7). The occurrence and incidence of complications were recorded.

## Inclusion and exclusion criteria

**Inclusion criteria were typical intermittent claudication with symptoms involving one lower limb; Discogenic low back pain, VAS score of low back pain > 3; Imaging findings suggest single segment degenerative lumbar spinal stenosis; The effect of standard conservative treatment for 3 months was not good; The operation method was PCLE-LIF**. The exclusion criteria were meyerding grade II and above slippage; Bilateral decompression is required; Severe osteoporosis; Revision surgery; Accompanied by peripheral nerve disease or joint disease or mental and psychological disease; No regular follow-up.

## Surgical procedure

All patients were intubated for general anesthesia, lying prone on the x-ray permeable operating table and body position pad, with the abdomen suspended to prevent excessive negative pressure. Adjust the lumbar bridge to expand the vertebral lamina space, and adjust the head and tail inclination of the operating table and the patient's body position through the C-arm fluoroscopy to ensure that the fluoroscopy result displayed is the standard lumbar anteroposterior and lateral position. After C-arm positioning and marking, complete routine disinfection and towel laying. The skin was incised with a sharp knife blade, and the pedicle puncture was performed using the pre-operative planned puncture route. After the puncture, a memory guide wire was placed, and the tail end of the guide wire was fixed on both sides of the operation area. Through the incision of implant nail, the myometrium was cut to 2 cm away from the central line of spinous process, and the muscle was passively separated and put into the step-by-step expansion tube and working channel. First, the soft tissues of the facet joint and the lower lamina margin were scraped under blind vision with a flat working channel, and the remaining soft tissues were removed with radiofrequency ablation electrodes and nucleus pulposus forceps to clearly expose the bony structures. Subsequently, a circular trephine or osteotome under the microscope was used to remove part of the inferior articular process. The upward resection range should reach the insertion point of ligamentum flavum, and the outward resection range should reach the upper articular process. Lamina rongeur or microscopical osteotome were used to gradually remove the upper articular process and caudal to the base of the upper articular process or the upper edge of the pedicle. The specific scope of bone structure resection is determined according to the operation space and decompression requirements. After the resection of the bony structure, the flat working channel was replaced by the oblique working channel to continue to complete the steps of intervertebral fusion. The long lingual surface of the oblique passage is used to protect the nerve, and the intervertebral space is treated under direct vision. The vertebral space is treated with lamina rongeur, reamer, scraper and curette, and the depth of the instrument into the intervertebral space is strictly limited. After intervertebral treatment, a trial model was placed into the intervertebral space to determine the size of the fusion cage. The bone grafting funnel is used to fill the intervertebral space with autologous bone particles. Then, the cage filled with autologous bone is implanted into the intervertebral space. The position of the cage is determined by C-arm fluoroscopy. The pedicle screw and connecting rod with appropriate length were implanted through the reserved track of memory guide wire, and the tail cap was placed and locked. After sufficient hemostasis, a drainage tube was placed and the wound was sutured layer by layer.

## Postoperative treatment

The drainage tube was pulled out when the drainage fluid was less than 50 ml on the first day after operation. If there was cerebrospinal fluid leakage, the time of pulling out the tube should be extended as appropriate. On the second day after operation, x-ray and three-dimensional CT of the lumbar spine were reexamined. If the internal fixation and cage position were satisfactory, the patients got out of bed with the assistance of lumbar brace. 4–5 days after operation, if no abnormal incision is observed, the patient can be discharged from the hospital and can resume standardized functional exercise. Three months after operation, if it is determined that the intervertebral fusion is good, remove the lumbar brace and perform normal lumbar movement.

## Statistical analysis

SPSS 20.0 statistical software was used for data analysis. The measurement data were expressed as mean ± standard deviation. The continuous data before and after operation were compared by paired *T*-test. The test level is taken from both sides *α* = 0.05.

## Result

A total of 62 patients were included in this trial, including 35 males and 27 females, aged 54.5 ± 12.0 years, and the duration of symptoms was 19.2 ± 13.1 months. All patients underwent PCLE-LIF operation, **including 35 cases of L4/5 and 27 cases of L5/S1**. All patients were assisted with posterior percutaneous pedicle screw fixation. The operation time was 128.2 ± 19.7 min and the hospital stay was 7.7 ± 1.4 days Table 1.

**Table 1:** Summary of the baseline data PCLE-LIF indicates percutaneous coaxial large-channel endoscopic lumbar interbody fusion; n indicates the total number of patients.

Characteristics	PCLE-LIF (*n* = 62)
**Age (years)**	54.5 ± 12.0
**Sex M/F**	35/27
**Duration of symptoms (months)**	19.2 ± 13.1
**Surgical location**	
L4/5	35
L5/S1	27
**Operating time (min)**	128.2 ± 19.7
**Hospital stay (days)**	7.7 ± 1.4

All the enrolled patients were followed up regularly for 1 year. The VAS scores of low back pain and lower extremity pain before operation were 4.48 ± 1.06 and 5.58 ± 0.98. The VAS scores of low back pain at 3 days, 3 months and 1 year after operation were 0.84 ± 0.81, 0.40 ± 0.56 and 0.39 ± 0.69, and the VAS scores of lower extremity pain were 0.91 ± 0.58, 0.31 ± 0.46 and 0.19 ± 0.40. The VAS scores at each follow-up time point after operation were significantly lower than those before operation (*p* < 0.05). The preoperative ODI score was 60.01 ± 6.21, and the postoperative 3 months and 1 year ODI scores were 15.58 ± 2.84 and 8.82 ± 2.15. The ODI scores at each follow-up time point were significantly lower than those before operation (*p* < 0.05). One year after operation, the results of macnab standard evaluation showed that 36 cases were excellent, 23 cases were good, 3 cases were fair, and 0 case was poor. The excellent and good rate was 95.2% Table 2. One year after operation, the fusion rate of the enrolled patients was 93.5%, including 4 cases of grade 3, 26 cases of grade 4 and 32 cases of grade 5 Table 3.

**Table 2:** Comparation of the VAS and ODI between pre and postoperative.

Characteristics	Pre-op	Post 3d-op	Post 3m-op	Post 1y-op
**VAS leg pain**	5.58 ± 0.98*	0.91 ± 0.58^/^	0.31 ± 0.46^/^	0.19 ± 0.40^/^
**VAS low back pain**	4.48 ± 1.06**	0.84 ± 0.81^%^	0.40 ± 0.56^%^	0.39 ± 0.69^%^
**ODI**	60.01 ± 6.21***		15.58 ± 2.84^@^	8.82 ± 2.15^@^

VAS indicates Visual Analogue Scale; ODI indicates Oswestry Disability Index; pre-op indicates preoperative; post-op indicates postoperative.

* *P* < 0.001 if / is compared with.

** *P* < 0.001 if % is compared with.

*** *P* < 0.001 if @ is compared with.

**Table 3:** Summary of interbody fusion rate and complications.

Characteristics	PCLE-LIF (*n* = 62)
**Degree of fusion rate**	
Level 1	0
Level 2	0
Level 3	4
Level 4	26
Level 5	32
Rate (%)	93.5%
**Complication**	
Numbness of lower limbs	2
Pain due to nerve edema	2
Cage immigration	1
Pedicle screw loosening	1
Dural sac tear	0
Infect	0
Rate (%)	9.6%

**Figure 1:**
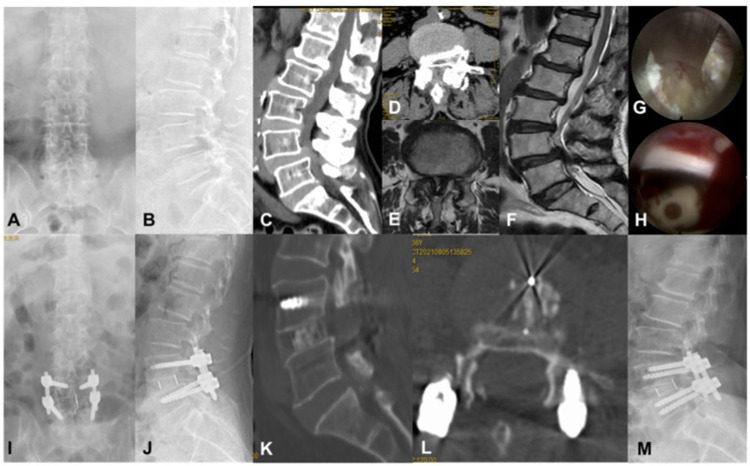
A 66-year-old female patient with discogenic low back pain and L4, 5 spinal stenosis. (**A–F**). Preoperative x-ray film, CT, and MRI showed L4, 5 intervertebral disc degeneration, ligamentum flavum hypertrophy, intervertebral disc herniation, and spinal stenosis; (**G, H**). The endplate was not damaged under the microscope during operation, and the position of the Cage and the range of decompression were satisfactory; (**I–L**). x-ray film and CT at 3 days after operation showed that the position of the Cage and the pedicle screw was satisfactory with bilateral screw rod fixation, and the decompression range was satisfactory; (**M**) x-ray film at 1 year after operation showed that the internal fixation was reliable and the intervertebral fusion was successful.

The incidence of postoperative complications was 9.6%. There were 2 cases of lower limb numbness on the operation side, of which 1 case occurred on the day after operation and 1 case occurred on the third day after operation. Both patients were relieved within 2 weeks after operation after nutritional nerve therapy. There were 2 cases of neuroedematous pain after operation, which occurred 2 days after operation. After pain relief and symptomatic treatment, they were relieved within 1 week after operation. One case of cage displacement and one case of pedicle screw loosening occurred after operation. Both patients were treated conservatively and interbody fusion was successful 3 months after operation Table 3 and Figure 1.

## Discussion

### Efficacy evaluation of PCLE-LIF

Percutaneous endoscopic lumbar fusion has developed rapidly in recent years. At present, there are a variety of surgical approaches and endoscopic systems for spinal minimally invasive surgeons to choose. Kim et al. Conducted a comparative study on the treatment of degenerative lumbar spinal stenosis by dual channel endoscopic fusion and minimally invasive trans foraminal lumbar fusion (MIS TLIF), and found that the two groups of patients can obtain good curative effects, with no difference in medium and long-term curative effects, and the former is better than the latter in early back pain relief (8). Ao et al. Compared the coaxial single channel endoscopic lumbar fusion with MIS TLIF and obtained similar results (9). These results confirm that the clinical effect of percutaneous endoscopic lumbar fusion is satisfactory. In recent years, with the improvement of instruments and surgical techniques, PCLE-LIF has also attracted the attention of spinal minimally invasive surgeons, but the relevant literature is less reported at present. In this group of cases, we applied PCLE-LIF to single segment lumbar spinal stenosis. The research results showed that the ODI and VAS scores after operation were significantly lower than those before operation, and the excellent and good rate of macnab was 95.2%. This result is similar to the previous research results using other endoscopic systems (10, 11), which also fully shows that PCLE-LIF is effective in the treatment of single segment lumbar spinal stenosis.

The percutaneous endoscopic fusion technique was questioned by many scholars because of its high complication rate in the early stage (2, 12). Even though the surgical instruments and techniques were improved later, it still had a high complication rate. A meta-analysis in recent years showed that the nerve injury rate of percutaneous endoscopic fusion technique was 3.3%–10% (13). In the early stage of percutaneous endoscopic lumbar fusion, the trans foraminal approach was often used, which led to a significant increase in the probability of travel root injury. In the retrospective study of a group of 25 patients with a total number of cases. In a retrospective study with a total of 25 patients and Nagahama operated with a small channel. There were 2 cases of nerve injury after operation, the incidence was 8%, and the symptoms were numbness in the root innervation area (14). In a retrospective study of a total of 30 patients and Morgenster operated with a large channel. There were 3 cases of travel nerve injury after operation, and the incidence was 10% (15). In this study, all patients had no travel root injury. This is because PCLE-LIF can effectively protect the travel root by using the intervertebral approach. However, there were 4 cases of walking nerve root stimulation in this group, with an incidence of 6%, which is similar to other previous studies on endoscopic lumbar fusion using other endoscopic systems (13). We believe that this is caused by insufficient resection of bony structures and narrow operation space in the early stage of operation, resulting in too much nerve pulling inward. In the later stage, after the improvement of operation, there was no walking root stimulation. In addition to nerve injury, fusion cage displacement and subsidence are also common complications (12). It is reported that endoscopic fusion may lead to poor endplate treatment due to the limitation of visual field and instruments, resulting in postoperative cage displacement and fusion failure (16). In this study, only one case of cage subsidence occurred, and the incidence rate was far lower than that in previous studies related to endoscopic lumbar fusion. However, the fusion rate of the enrolled cases was 93.5% one year after operation, which was similar to the previous research results of lumbar fusion using other surgical methods (17, 18). These results suggest that the fusion effect of PCLE-LIF is satisfactory. We believe that this is because PCLE-LIF has obvious operational advantages over previous small channel endoscopic systems. Because the large channel endoscopic system can allow the conventional open surgery to participate in the endoscopic operation, which effectively improves the operation efficiency and intervertebral treatment efficiency. The full-size cage model used during the operation also ensures the contact area between the cage and the endplate. In addition, the micro adjustment and expansion method of the expandable reamer can protect the endplate to the greatest extent from damage.

### Key points of PCLE-LIF operation

We summarize the operation skills during the operation, which we believe will be helpful for the development of this technology. First, the pretreatment of intraoperative bleeding. Due to the expansion of channel channel and visual field, intraoperative bleeding is more common in the large channel endoscopic system than in the conventional small channel. Our experience is that four three liter bags are simultaneously connected to the endoscope to increase the intraoperative water pressure and control bleeding. In addition, preoperative intravenous use of tranexamic acid can also effectively prevent intraoperative bleeding. Second, select the appropriate incision to place the working channel. Because the channel size of PCLE-LIF is 10.2 mm inner diameter and 11.2 mm outer diameter, we use the incision of implant nail to place the working channel to reduce skin damage. Third, the use of the flat mouth channel. During the PCLE-LIF operation, we all use the flat mouth channel for the operation outside the spinal canal. Its role lies in the following two points. First, before placing the endoscope, we use the flat mouth channel to scrape out the soft tissue of the facet joint and the lower edge of the upper vertebral lamina under blind vision, so as to reduce the time for processing the soft tissue under the microscope. Second, after placing the endoscope, The good sealing performance of the flat mouth channel can effectively block the surrounding soft tissue from entering the operation field, thus affecting the operation. Therefore, we choose to complete the resection of bone structures with the assistance of the flat mouth channel, which can effectively reduce the operation time. Fourth, the use of bone knife and circular saw under the microscope. One of the key points to improve the efficiency of PCLE-LIF surgery is the rapid removal of bone structures. Bone knife and circular saw under the microscope allow us to accurately remove bone structures under visual conditions. Our experience is that the bony structure in the target area is removed with a circular saw under the microscope, and then the bony structure in the target area is taken out by sections with a bone knife under the microscope. The resection range is determined according to the actual needs. During the resection process, the depth and direction should be strictly controlled to avoid nerve damage. Fifthly, pretreatment of the insertion point of the annulus fibrosus. Before intervertebral treatment, we suggest to use blue forceps and radiofrequency ablation electrodes to fully remove the attachment part of the annulus fibrosus and clearly expose the bony structure of the upper and lower vertebral margins, which will help us to judge the depth and scope of intervertebral treatment. Sixthly, the application of visual curet. During intervertebral treatment, after the conventional curet, scraper and reamer are scraped out, some soft tissues are often left in the endplate, resulting in repeated intervertebral operations under blind vision. The application of visual curet can accurately remove the residual soft tissue on the endplate, effectively reduce the surgical procedures and improve the surgical efficiency.

## Conclusion

Percutaneous coaxial large-channel endoscopic lumbar interbody fusion in the treatment of degenerative lumbar spinal stenosis has good short-term efficacy and high safety, and is worthy of popularization.

## Data Availability

The original contributions presented in the study are included in the article/Supplementary Material, further inquiries can be directed to the corresponding author/s.
